# Imaging of Indocyanine Green-Human Serum Albumin (ICG-HSA) Complex in Secreted Protein Acidic and Rich in Cysteine (SPARC)-Expressing Glioblastoma

**DOI:** 10.3390/ijms24010850

**Published:** 2023-01-03

**Authors:** Hye Jung Jang, Myung Geun Song, Cho Rong Park, Hyewon Youn, Yun-Sang Lee, Gi Jeong Cheon, Keon Wook Kang

**Affiliations:** 1 Department of Nuclear Medicine, Seoul National University College of Medicine, Seoul 03080, Republic of Korea; 2 Department of Biomedical Sciences, Seoul National University Graduate School, Seoul 03080, Republic of Korea; 3 Cancer Research Institute, Seoul National University College of Medicine, Seoul 03080, Republic of Korea; 4 Biomedical Research Institute, Seoul National University Hospital, Seoul 03080, Republic of Korea; 5 Cancer Imaging Center, Seoul National University Hospital, Seoul 03080, Republic of Korea; 6 Institute of Radiation Medicine, Medical Research Center, Seoul National University Hospital, Seoul 03080, Republic of Korea

**Keywords:** glioblastoma, fluorescence-guided surgery, secreted protein acidic and rich in cysteine (SPARC), human serum albumin, indocyanine green

## Abstract

Glioblastoma is the most common and fatal primary glioma and has a severe prognosis. It is a challenge for neurosurgeons to remove brain tumor tissues completely by resection. Meanwhile, fluorescence-guided surgery (FGS) is a technique used in glioma surgery to enhance the visualization of tumor edges to clarify the extent of tumor resection. Indocyanine green (ICG) is the only FDA-approved NIR fluorescent agent. It non-covalently binds to human serum albumin (HSA). Secreted protein acidic and rich in cysteine (SPARC) is an extracellular glycoprotein expressed in gliomas and binds to albumin, suggesting that it plays an important role in tumor uptake of the ICG-HSA complex. Here we demonstrate the binding properties of HSA or SPARC to ICG using surface plasmon resonance and saturation binding assay. According to in vitro and in vivo studies, the results showed that the uptake of ICG-HSA complex was higher in SPARC-expressing glioblastoma cell line and tumor region compared with the uptake of free ICG. Here, we visualized the SPARC-dependent uptake of ICG and ICG-HSA complex in U87MG. Our results demonstrated that the ICG-HSA complex is likely to be used as an efficient imaging agent targeting SPARC-expressing tumors, especially glioblastoma.

## 1. Introduction

Glioblastoma is the most lethal brain cancer in adults and is characterized by its inevitably recurrent and poor prognosis. Because most glioblastoma patients die of their disease within one year, none have long-term survival [1]. Therefore, accurate diagnosis and prediction of prognosis are very important [2]. A surgical resection is a common approach to brain cancer treatment. Because tumor resection is based on macroscopic identification of the tumor region, it is challenging for complete resection and clearance of tumor tissues [3,4]. Optical imaging techniques for surgery can identify edges containing intraoperative tumors and their microscopic ranges to generate real-time data that improve surgical decisions without significantly extending surgical time [5].

Fluorescence-guided surgery (FGS) uses an imaging agent to provide intraoperative contrast, allowing surgeons to identify tumor cells that might otherwise be missed. Currently, there are clinical studies based on fluorescent contrast agents such as 5-aminolevulinic acid (ALA), fluorescein, and indocyanine green (ICG) [6,7]. The 5-ALA, approved by the European Medicines Agency (EMA) for FGS, has been used for the surgical treatment of gliomas [8]. However, FGS using 5-ALA in glioma resection has a major limitation of inconsistency [9]. ICG and fluorescein are the only fluorescence contrast agents approved by the U.S. Food and Drug Administration (FDA) and have an excellent safety profile for clinical applications [10]. On the other hand, many fluorophores for imaging other body regions are not approved for use in the brain because they are mutagenic [5]. Fluorescein is used for diagnostic angiography or vascular endoscopy of the retina and iris vasculature. ICG is used to measure cardiac output, liver blood flow, and hepatic function and for ophthalmic angiography [11,12,13,14].

Among the various fluorescent agents, ICG is a biocompatible near-infrared (NIR) fluorophore with an emission peak of around 800 nm [15,16]. Near-infrared fluorescence imaging of ICG has been actively investigated, including image-guided surgery, because of advantages such as its simplicity, high sensitivity, safety, and fast imaging speed [17,18]. In vivo imaging in the near-infrared range is effective because NIR light has a much lower tissue absorption coefficient and thus can increase tissue penetration [19]. Additionally, low background noise and low NIR fluorescence scattering decrease auto-fluorescence compared with visible light. It is beneficial to increase molecular sensitivity [20]. However, ICG has a very short blood half-life, low photostability, poor solubility in aqueous media, and a strong tendency to form aggregates, which strongly limits its use [21].

ICG is known to prefer reversibly binding to serum albumin, and highly protein-bound ICGs behave like macromolecules in blood circulation [10]. Human serum albumin (HSA) is the most abundant protein present in human blood [22]. It has been used as a versatile tool for drug delivery due to its circulating half-life of approximately 19 days [23] and advantages for clinical applications such as water-solubility, stability, and non-toxicity [24,25]. In addition, HSA has an affinity for ICG through non-covalent interactions [18] and consequently increases the fluorescence quantum yield [26,27]. Therefore, we can expect that the use of the ICG-HSA complex can improve the circulation time of ICG in the blood and its delivery efficiency to the target, as well as enhance the fluorescence intensity.

It has been reported that the albumin-binding proteins in the tumor endothelium (gp60 receptor, albondin) and secreted SPARC protein in the tumor stroma have a high binding affinity for albumin, thereby enhancing the retention of albumin in the tumor region [28,29,30]. In particular, as SPARC overexpression is associated with malignancy in brain tumor cells [31,32,33], the use of albumin could be expected to take advantage of the presence of SPARC to increase ICG accumulation in tumors. Here, we investigate the potential of the ICG-HSA complex as an imaging agent for visualizing SPARC-expressing glioblastoma by NIR imaging.

## 2. Results

### 2.1. Binding ICG to HSA and SPARC

**Figure 1:**
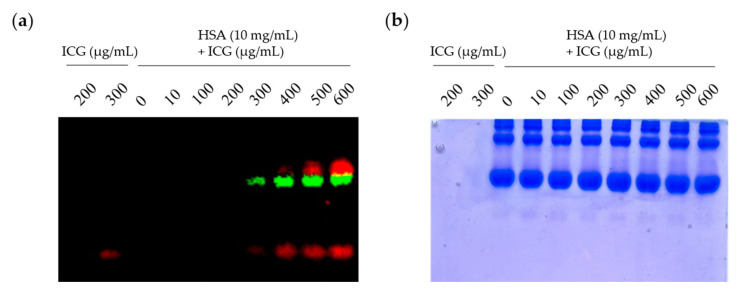
Binding ICG to HSA (**a**) Optical imaging of ICG and ICG-HSA complex. Saturation binding analysis using Native PAGE gel electrophoresis in a mixture of HSA and ICG at various concentrations. After the fluorescence signal was unmixed by Lumina II Imaging System, the ICG-HSA complex was presented as green, and the unbound ICG was presented as red; (**b**) image of Native PAGE gel after Coomassie blue staining.

Albumin has a binding pocket to which various small molecules can be non-covalently attached [24,25]. To confirm that ICG could non-covalently attach to the albumin surface, the saturation binding assay using Native PAGE gel was used. In the fluorescence image (Figure 1a) and Coomassie blue staining results (Figure 1b), a fluorescence signal (green) was observed in an albumin-sized protein band. Free ICG confirmed the signal (red) at the bottom of the gel. Measurements of the ICG-HSA complex using saturation binding assays do not offer insights regarding the kinetics of binding. Therefore, we further investigate the properties of ICG for HSA and SPARC using surface plasmon resonance (SPR) analysis (Table 1 and Figure S1). The association rate (K_a_) and dissociation rate (K_d_) of ICG and albumin were 193 and 0.00792, respectively, and the K_D_ value was 41.1 μM. Interestingly, ICG also had a binding affinity for SPARC. Its association rate (K_a_) and dissociation rate (K_d_) were 126 and 0.00471, respectively, and the K_D_ value was 37.3 μM. These results indicate that ICG can bind HSA as well as to SPARC.

### 2.2. Uptake of ICG-HSA Complex In Vitro

In a previous study, the presence of the SPARC protein affected the intracellular uptake of albumin [29]. The expression level of SPARC was confirmed by real-time PCR and Western blot analysis in various cancer cell lines (Figure S2a,b). To confirm the difference in the cellular uptake of ICG-HSA, two U87MG cell lines with different SPARC expressions were used: U87MG cells overexpressing SPARC protein and U87MG-shSPARC cells knocking down protein expression of SPARC.

**Figure 2:**
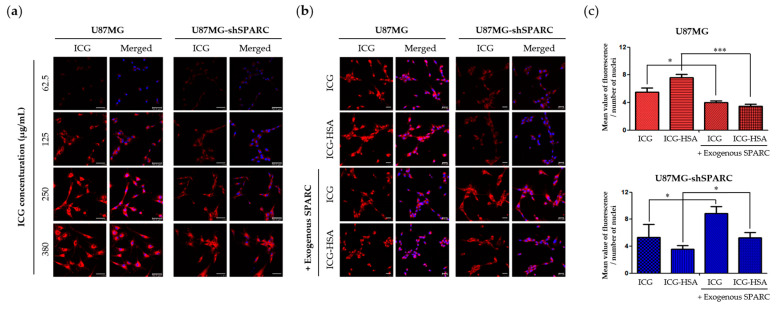
In vitro fluorescence imaging of ICG-HSA complex in U87MG and U87MG-shSPARC cell lines. (**a**) Fluorescence images of U87MG and U87MG-shSPARC cells after incubation with different concentrations of ICG, respectively. Scale bars, 25 μm; (**b**) fluorescence images of U87MG and U87MG-shSPARC cells after incubation with ICG or ICG-HSA complex. The ICG is red, and the DAPI-stained nuclei are blue. Scale bars, 50 μm. (**c**) Quantifying ICG or ICG-HSA uptake in each cell type from confocal images *: *p* < 0.05, ***: *p* <0.001.

To compare the uptake rate of ICG in the two cell lines with different SPARC protein expressions, the fluorescence signal was increased in both cell lines depending on the concentration of ICG treatment (Figure 2a). However, the fluorescence signal from ICG was stronger in U87MG than in U87MG-shSPARC. To confirm the importance of SPARC protein in cellular uptake of ICG or ICG-HSA complex, ICG or ICG-HSA complex (molar ratio ICG:HSA = 1:0.5) was treated in two cell lines, and the fluorescence signal was observed. As a result, the highly accumulated fluorescence signal of ICG or ICG-HSA complex was detected in U87MG compared to U87MG-shSPARC (Figure 2b,c). When the exogenous SPARC protein was treated under these conditions, it was observed that the intracellular uptake of ICG and ICG-HSA was restored in U87MG-shSPARC. These results indicate that SPARC plays an important role in the uptake process of ICG-HSA and ICG.

### 2.3. In Vivo and Ex Vivo Fluorescence Imaging of ICG-HSA Complex

**Figure 3:**
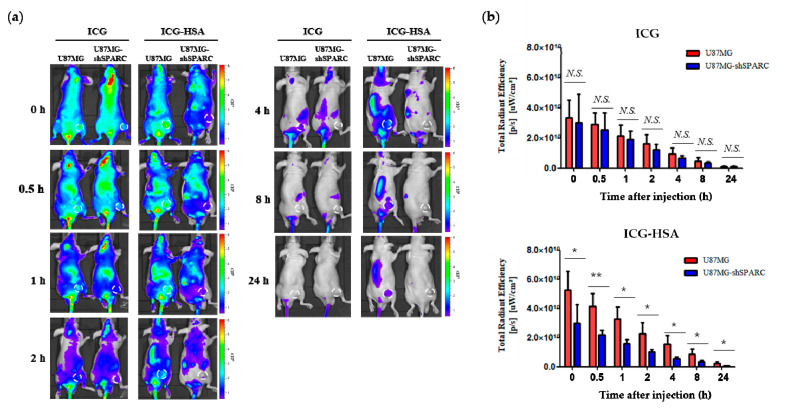
In vivo fluorescence imaging of ICG-HSA complex in U87MG and U87MG-shSPARC tumor-bearing mice. (**a**) Fluorescence images at different time points; 0, 0.5, 1, 2, 4, 8, and 24 h. The white circle indicates the tumor region; (**b**) NIR fluorescence intensity of the tumor region. Fluorescence signals from tumors were acquired through ROI analyses (n = 3). N.S. non-significant difference. *: *p* < 0.05, **: *p* < 0.01.

The fluorescence imaging of ICG-HSA was investigated in xenograft tumor mouse models using U87MG and U87MG-shSPARC cells. In the subcutaneous xenograft models, ICG or ICG-HSA complex was injected through the tail vein. The whole-body fluorescence signal was measured using the NIR fluorescence imaging system for up to 24 h (Figure 3a). ICG-HSA showed a stronger fluorescence signal in the U87MG tumor than in the U87MG-shSPARC tumor. On the other hand, there was no significant difference in fluorescence intensity in both tumors in the group treated with ICG alone (Figure 3b).

**Figure 4:**
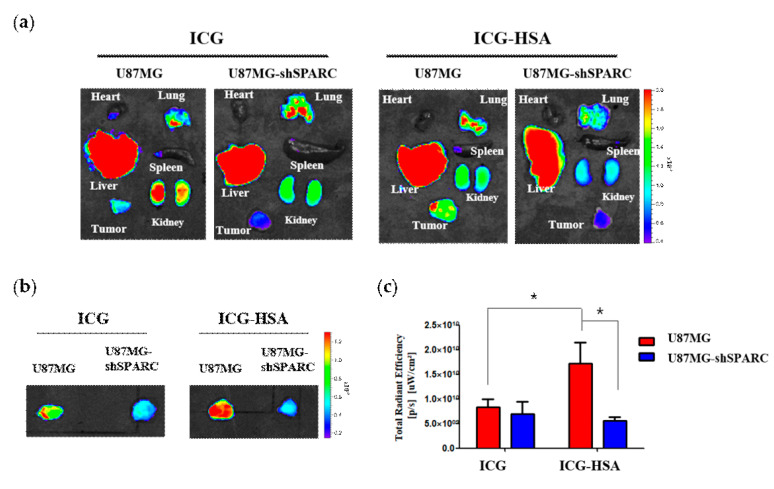
Ex vivo fluorescence imaging of ICG-HSA complex in U87MG and U87MG-shSPARC tumor models. (**a**) Ex vivo fluorescence images of tumors and organs 1 h after the injection of ICG or ICG-HSA; (**b**) ex vivo fluorescence images of tumors with 10 sec exposure time; (**c**) fluorescence intensity of tumors region. *: *p* < 0.05.

In ex vivo fluorescence images of organs and tumors dissected 1 h after injection, most of the free ICG and ICG-HSA were observed in the liver, lungs, and kidneys (Figure 4a). On the other hand, we confirmed that an enhanced fluorescence signal could be detected in U87MG tumors of ICG-HSA-treated mice (Figure 4a,b). In the group treated with ICG, an average of about 1.2 times stronger fluorescence signal was observed in U87MG tumors than in U87MG-shSPARC tumors. In ICG-HSA, the signal from U87MG tumors was about 3.1 times higher than that of U87MG-shSPARC tumors. Compared with free ICG-treated mice, U87MG tumors of ICG-HSA-treated mice exhibited significantly higher fluorescence intensity, approximately 2.1-fold at 1 h post-injection. On the contrary, in U87MG-shSPARC, the ICG-treated group showed a slightly higher fluorescence signal (about 1.2-fold) than the ICG-HSA-treated group (Figure 4c).

### 2.4. Fluorescence Imaging of ICG-HSA Complex and SPARC in Tumor Tissue

**Figure 5:**
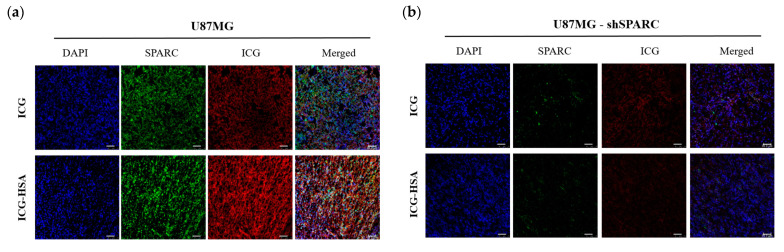
Fluorescence imaging of ICG-HSA complex in tumor tissue. Confocal images of SPARC obtained through immunofluorescence staining and injected ICG were confirmed in frozen tumor sections. (**a**) Fluorescence images in the U87MG; (**b**) U87MG-shSPARC tumor. Scale bars, 50 μm.

We confirmed the localization of the SPARC protein and the ICG fluorescence signal in the resected tumor. Each tumor was extracted 1 h post-injection of each fluorescent probe, and immunostaining was performed to observe the location of the SPARC protein. Positive fluorescence signals of ICG and SPARC protein were detected in U87MG tumor sections (Figure 5a) but not in U87MG-shSPARC tumor sections (Figure 5b). In addition, in the U87MG tumor section, ICG-HSA showed a stronger fluorescence signal than ICG alone.

## 3. Discussion

It is important to accurately decide where the tumor margin is in tumor removal surgery to reduce the risk of reoperation and recurrence. Fluorescence-guided surgery (FGS) enables real-time visualization of the tumor edge in figuring out the extent of tumor resection in glioma surgery, providing decisive guidance to surgeons.

ICG, one of the clinically available dyes, is a NIR contrast agent with attractive features of low toxicity, high light absorption, and intensive fluorescence [16]. However, ICG has a very short blood half-life of fewer than 5 min in humans, poor photostability and solubility, and easy aggregate formation, which strongly limits its use [21].

Human serum albumin (HSA), to which ICG can be non-covalently attached, is an in vivo nanocarrier known to increase the circulation time of small molecules in the blood. HSA is a versatile tool for drug delivery due to its long circulating half-life in blood and advantages for clinical applications such as water-solubility, stability, and non-toxicity [24,25]. This study verified the possibility of selectively accumulating in U87MG by using a non-covalently bound HSA and ICG complex as an imaging probe for FGS.

Accumulating macromolecules (>40 kDa) within the tumor interstitium is known to be due to enhanced permeation and retention effects. Due to this effect, it is known that albumin (about 67 kDa) leaks through the loose blood vessels around the tumor and accumulates in the tumor interstitium. Moreover, tumors actively uptake albumin as an energy source to accelerate growth by breaking it down into its component amino acids in lysosomes [23].

Another mechanism for intratumoral accumulation of albumin is also known to involve receptor-mediated albumin uptake pathways by albumin-binding proteins such as “secretory proteins, acid- and cysteine-rich secreted proteins” (SPARC) [34]. In a previous study, we confirmed the binding of SPARC to albumin in vitro and validated the SPARC-mediated targeting potential of albumin in vivo [29]. In this study, the binding affinity of ICG to HSA was observed using Native PAGE gel (Figure 1a,b) and SPR analysis (Table 1 and Figure S1). Interestingly, it was newly revealed that ICG had a binding affinity with HSA and SPARC. Here, we confirmed the possibility that ICG can bind to both HSA and SPARC, respectively, so these complexes may be ingested into tumor cells. In vitro uptake of ICG or ICG-HSA complex was restored by treatment of exogenous SPARC protein in U87MG-shSPARC cells (Figure 2b). The above reasons may explain the important role of the SPARC protein in the mechanism of selective accumulation of both ICG and ICG-HSA complex in brain tumor models.

In the case of brain tumors, it has been reported that the expression of the SPARC protein is involved in the progression of malignant tumors [30,31]. In our in vivo study, the NIR fluorescence intensity of the tumor region was higher in the SPARC-positive U87MG tumor than in the U87MG-shSPARC tumor (Figure 3 and Figure 4). Therefore, when performing fluorescence-guided surgery based on the SPARC target ICG-HSA complex in malignant brain tumors, it is expected to be a promising solution for surgeons by making it possible to identify the SPARC-positive region of cancer.

It has been reported that ICG shows weak fluorescence only in the near-infrared region in the free (unbound) state in a dilute aqueous solution [35]. Our results showed higher fluorescence intensity in ICG mixed with HSA than in ICG alone (Figure S3). The fluorescence signal of ICG-HSA (Ex = 780 nm, Em = 845 nm) was about 3.4 times as high as that of ICG alone using an in vivo imaging system. The fluorescence signal was strongest when the molar ratio of ICG and HSA was 1:0.13 to 1:0.54. Due to the characteristics of ICG, it may be possible that fluorescence signals were amplified in U87MG in our animal imaging results. However, comparing the groups treated with ICG and ICG-HSA, fluorescence signals increased only in U87MG, and U87MG-shSPARC did not increase. It could be explained by the amplification of the ICG fluorescence signal by HSA, but more than that, it could be evidence of its intratumoral accumulation effect by SPARC.

ICG also represents concentration-dependent fluorescent quenching (self-quenching) [36,37,38]. However, in our results, the starting point of self-quenching was different in free ICG (62.5 μg/mL) and HSA-mixed ICG (125 μg/mL). It means that HSA could prevent the self-quenching of the ICG. In addition, the intensity of the fluorescent signal could vary depending on the composition of the aqueous solution in which ICG is present. Therefore, it is essential to define the optimal molecular density of ICG and HSA, representing the maximum fluorescence intensity per ICG molecule.

To allow the ICG to circulate in the blood for a more extended period, the ICG may include an additional albumin-binding portion [39], and the ICG and albumin may be covalently bonded [40]. The albumin ICG conjugate has the advantage of increasing circulation time in the blood with a stable binding. Still, the cost and side effects of the conjugated agent should be considered. Since HSA and ICG used in this study are already sold commercially alone, they can be used immediately as needed at the surgical site and are more likely to avoid side effects issues than conjugates by other chemical covalent bonds. In addition, since preoperative serum albumin levels in patients with malignant brain tumors could positively affect the survival curve [41], it can be assumed that injecting exogenous albumin into the body can help the patient’s postoperative recovery as well as effective FGS progression.

Overall, we evaluated the potential of the ICG-HSA complex as a valuable fluorescence agent to improve FGS outcomes for glioblastoma patients by providing accurate resection of tumor margins via SPARC targeting of the tumor. In conclusion, the ICG-HSA complex can be used as a NIR imaging agent for visualizing SPARC-expressing glioblastoma, which will be helpful for FGS.

## 4. Materials and Methods

### 4.1. Cell Lines

Human glioma cells (U87MG) were obtained from American Type Culture Collection (ATCC, Manassas, VA, USA). Low SPARC-expressing U87MG cells, U87MG-shSPARC, were established from a previous study [29]. Minimum Essential Medium (MEM; Gibco, Grand Island, NY, USA) contains 10% (*v/v*) fetal bovine serum (FBS; Gibco) and 1% antibiotics containing penicillin/streptomycin (Invitrogen, Carlsbad, CA, USA). Cells were incubated in a 37 °C humidified incubator with a 5% CO_2_ atmosphere.

### 4.2. Saturation Binding Assay

Both ICG and HSA were dissolved in distilled H_2_O. In a volume of 10 μL, 100 μg of HSA (MP biomedicals, Irvine, CA, USA) was mixed with different amounts of ICG (ranging from 0.019 to 5 μg). After incubation for 30 min at room temperature (RT), 10 μL of electrophoresis sample buffer 2× non-reducing (sc-45085; Santa Cruz Biotechnology, Dallas, TX, USA) was added to each vial. Then, the samples were loaded into Native PAGE gel for electrophoresis. The gel was imaged with Lumina II optical imaging system (PerkinElmer, Waltham, MA, USA). The fluorescence signal was unmixed on the different spectrums of the ICG and ICG-HSA complexes.

### 4.3. Western Blot

Cells were lysed in 4 °C conditions, using radio-immunoprecipitation assay (RIPA) buffer (Sigma-Aldrich, St. Louis, MO, USA) and protease inhibitor cocktail (Roche, Nutley, NJ, Switzerland). Supernatants were collected after centrifugation at 15,000 rpm for 20 min at 4 °C. Protein concentrations were measured by a BCA protein assay kit (Thermo Fisher Scientific, Waltham, MA, USA). Total protein (15 μg) mixed with 4× polyacrylamide gel electrophoresis sample buffer and 10x sample reducing agent (Invitrogen, Carlsbad, CA, USA) was loaded onto 12% SDS-PAGE gel. After gel electrophoresis, the proteins were transferred onto polyvinylidene difluoride (PVDF) membranes (Millipore, Billerica, MA, USA). The PVDF membranes were blocked with 5% BSA in Tris-buffered saline containing Tween-20 (TBST) for 1 h at RT. Then the membranes were incubated overnight at 4 °C with primary antibody for SPARC (#5420S; Cell Signaling Technology, Danvers, MA, USA; diluted 1:2000) and β-actin (A5441; Sigma-Aldrich, St. Louis, MO, USA; diluted 1:5000). The membranes were then probed with HRP-conjugated anti-rabbit or anti-mouse secondary IgG (Cell Signaling Technology). Proteins were detected with an enhanced chemical luminescence detection reagent (Roche). The signal intensities were measured using a LAS-3000 imaging system (Fujifilm, Tokyo, Japan)

### 4.4. Real-Time PCR

Total RNA was isolated from cells (U87MG, U87MG-shSPARC, PC3, MDA-MB 231, A549) using Trizol reagent (Invitrogen) according to the manufacturer’s protocol. Reverse transcription was performed with 2 μg of total RNA using amfiRivert Platinum cDNA synthesis Master Mix (GenDEPOT, Barker, TX, USA). From synthesized cDNA, the mRNA expression level of SPARC and GAPDH were detected using TB GreenTM Premix EX TaqTM (#RR420A; TAKARA Bio Inc., Kusatsu, Japan) and analyzed by ABI 7300 Real-Time PCR system. The sequences of the forward and reverse primers of SPARC were 5′-GG TTC AAA CTT TTG GGA GCA-3′ and 5′-CC GAT TCA CCA ACT CCA C-3′. In addition, the sequences of the forward and reverse primers of GAPDH were 5′- TG CAC CAC CAA CTG CTT AGC 3′ and 5′-GG CAT GGA CTG TGG TCA TGA G-3′.

### 4.5. Surface Plasmon Resonance (SPR) Analysis

HSA or SPARC protein was immobilized on a carboxymethyl dextran sensor (CMDH) chip (#13206066; Reichert Technologies, Buffalo, NY, USA). ICG was injected as an analyte with an association time of 3 min and a dissociation time of 5 min in a 2-fold dilution series ranging from 500 to 15.625 μM at a constant flow rate of 20 μL/min. A 1:1 Langmuir kinetic fit was applied to obtain the association rate constant K_a_, dissociation rate constant K_d_, and the dissociation constant K_D_. These rates were measured by SPR spectrometry (SR7500DC; Reichert Technologies).

### 4.6. In Vitro Fluorescence Imaging

Human glioblastoma cell lines, U87MG and U87MG-shSPARC, were seeded on cover-glass in a 12-well plate (Nalge NUNC International, Rochester, NY, USA; 1 × 10^5^ cells/well), respectively. Cells were incubated with ICG or ICG-HSA complex for 30 min and then washed three times with PBS. Cell fixation by paraformaldehyde (Santa Cruz Biotechnology, Inc.; 300 μL, 10 min/well) and mounting with ProLongTM Gold antifade reagent with DAPI (Invitrogen) and covered the samples with a cover slide. Fluorescence signals were detected using a confocal laser scanning microscope (Leica TCS SP8; Leica, Wetzlar, Hesse, Germany) in the specific range of wavelength (DAPI; 401–480 nm, ICG; 633–800 nm). Fluorescence intensities were analyzed using a LAS X system (Leica Microsystems; Leica).

### 4.7. In Vivo Fluorescence Imaging and Biodistribution

Six-week-old male BALB/c nude mice were obtained from Orient Bio, Inc. (Seongnam, Korea). All experiments were approved by the Institutional Animal Care and Use Committee of Seoul National University Hospital (SNUH-IACUC, No. 15-0279). To set up tumor xenograft models, U87MG and U87MG-shSPARC cell lines were injected subcutaneously into the thigh leg, 5x10^6^, respectively. About 2 weeks later, a tumor was formed in the thigh leg, and tumor size was measured by a caliper. The tumor-bearing mice were injected intravenously with 150 μg of ICG (7.5 mg/kg) or ICG-HSA complex (molar ratio of 1:0.5), respectively. After 0, 0.5, 1, 2, 4, 8, and 24 h, fluorescence signals were obtained by Lumina II (Perkin Elmer, Waltham, MA, USA). After 1 h, the mice were sacrificed, and the fluorescence signal intensities of the heart, liver, spleen, lung, kidney, and tumor were measured using Living Image software (version 2.5).

### 4.8. Immunofluorescence Staining

Tumors were harvested from sacrificed mice 1 h after ICG or ICG-HSA injection and embedded in optimal cutting temperature (OCT) compound (Leica biosystems, Wetzlar, Hesse, Germany). After freezing at −80 °C for 24 h, the specimens were cut into 8 μm sections. Frozen tumor slides were thawed for 5 min at RT, followed by permeabilization of samples with acetone at −20 °C for 10 min and washing the slides three times with PBS for 5 min. After the permeabilization step, the samples were blocked with 5% BSA in PBS for 1 h at RT. After blocking, the samples were treated with anti-SPARC primary antibody (#AF941; R&D systems, Minnneapolis, MN, USA; diluted 1:10) in 0.5% BSA and incubated at 4 °C overnight. On the following day, the samples were incubated with Alexa Fluor^TM^488 donkey anti-goat IgG secondary antibody (A11055; Invitrogen; diluted 1:400) in 0.5% BSA for 2 h at RT. After incubation, the samples were washed three times with PBS for 5 min and mounted with ProLong^TM^ Gold antifade reagent with DAPI (Invitrogen). Fluorescence signals were obtained by confocal laser scanning microscope (Leica TCS SP8; Leica).

### 4.9. Statistical Analysis

Quantitative data were expressed as mean ± standard deviation. Means were compared using the Student’s t-test provided by Excel 2013 (Microsoft Corporation, Redmond, WA, USA) or GraphPad Prism version 5.0 (GraphPad Software Inc., San Diego, CA, USA). A *p*-value of less than 0.05 was considered statistically significant.

## 5. Conclusions

In this study, it was confirmed that ICG could be attached to SPARC and HSA in vitro, and ICG and ICG-HSA were absorbed into the cells in a SPARC-dependent manner. In in vivo images, ICG-HSA was found to have a stronger fluorescence signal in SPARC-positive tumors than free ICG. In conclusion, we confirmed the potential of ICG-HSA as a SPARC targeting agent that could be helpful for FGS.

## Figures and Tables

**Table 1:** Surface Plasmon Resonance (SPR) analysis.

Ligands	k_a_ (1/Ms)	k_d_ (1/s)	K_D_ (μM)
Human Serum Albumin	193	0.00792	41.1
SPARC	126	0.00471	37.3

## Data Availability

Not applicable.

## Supplementary Materials

The following supporting information can be downloaded at: https://www.mdpi.com/article/10.3390/ijms24010850/s1.
