# Supplementary material for: Imaging of Indocyanine Green-Human Serum Albumin (ICG-HSA) Complex in Secreted Protein Acidic and Rich in Cysteine (SPARC)-Expressing Glioblastoma
Source: Int J Mol Sci. 2023 Jan 3;24(1):850. doi: 10.3390/ijms24010850 (PMC9821702; doi:10.3390/ijms24010850)
Supplement: Supplementary file 1 [file ijms-24-00850-s001.zip › ijms-2034450-supplementary.pdf]

Supplementary Material

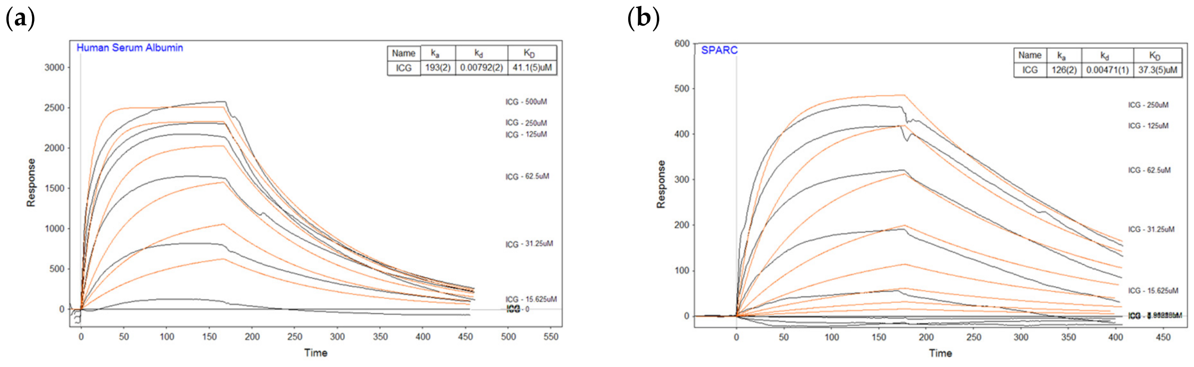

Figure S1. Surface Plasmon Resonance (SPR) analysis of ICG binding to HSA or SPARC. Plots illustrating the experimental curve-fitting methodology. Association and dissociation phases can be seen in each plot. A range of concentrations of the analyte (0, 15.625, 31.25, 62.5, 125, 250, or 500 µM of ICG) was used for the kinetic evaluation. (a) HSA was used as a ligand; (b) SPARC was used as a ligand.

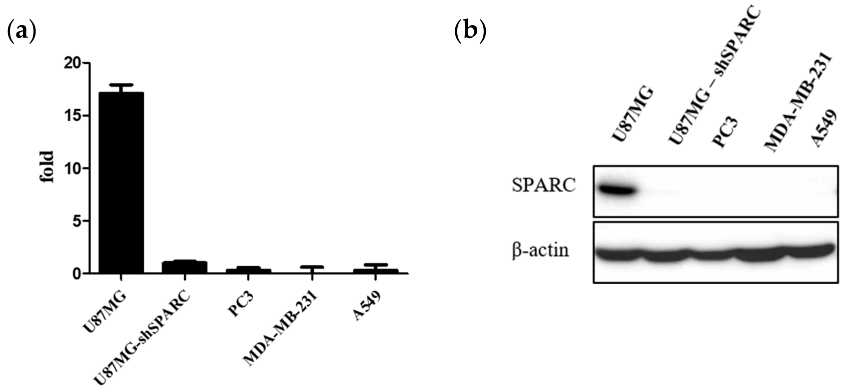

Figure S2. SPARC expression in tumor cell lines. (a) Real-time PCR for mRNA expression level analysis; (b) Western blot for protein expression level analysis.

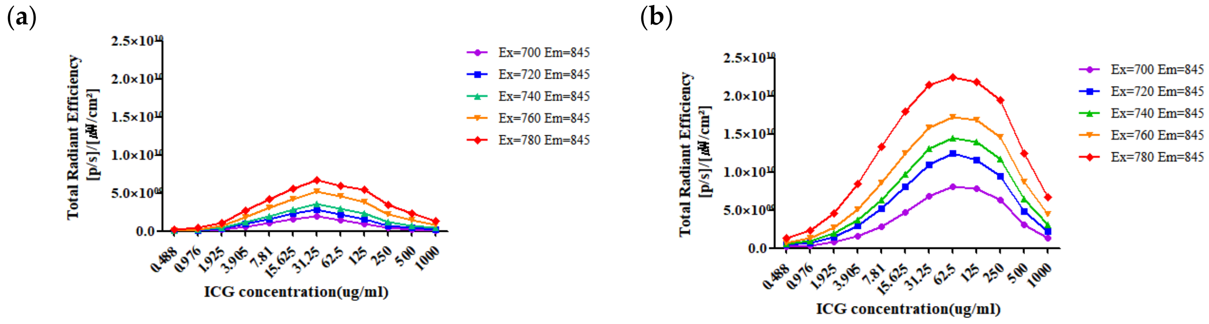

Figure S3. Fluorescence signal intensity of ICG and ICG-HSA complex in suspension. In a black 96-well plate, the intensity of the fluorescence signal was measured at different excitation wavelengths (700, 720, 740, 760, and 780 nm) and emission wavelength 845 nm. (a) Serially diluted free ICG (0.488 to 1000 µg/mL); (b) diluted free ICG with HSA (20 mg/mL)
